# *In vivo* Imaging of a Novel Strain of *Bacteroides fragilis* via Metabolic Labeling

**DOI:** 10.3389/fmicb.2018.02298

**Published:** 2018-10-01

**Authors:** Wenye Xu, Peizhu Su, Lijun Zheng, Hongying Fan, Ye Wang, Yangyang Liu, Yuqing Lin, Fachao Zhi

**Affiliations:** ^1^Guangdong Provincial Key Laboratory of Gastroenterology, Department of Gastroenterology, Institute of Gastroenterology of Guangdong Province, Nanfang Hospital, Southern Medical University, Guangzhou, China; ^2^Department of Gastroenterology, First People’s Hospital of Foshan Affiliated to Sun Yat-sen University, Foshan, China; ^3^Guangzhou Zhiyi Biotechnology Co., Ltd., Guangzhou, China; ^4^Guangdong Provincial Key Laboratory of Tropical Disease Research, School of Public Health, Southern Medical University, Guangzhou, China

**Keywords:** *Bacteroides fragilis*, ZY-312, probiotic, colonization, *in vivo* imaging

## Abstract

Non-toxigenic *Bacteroides fragilis* is regarded as a potential candidate for probiotic owing to its various advantages. We previously isolated a new strain of *B. fragilis* (ZY-312) and verified its biosafety and capability of inhibiting the growth of pathogens *in vivo*. However, the colonization of ZY-312 in gastrointestinal (GI) tract remains to be determined. To track the colonization of ZY-312, mice were gavaged with ZY-312 labeled by means of metabolic oligosaccharide engineering and bioorthogonal click chemistry or given AF647-dibenzocyclooctyne (DIBO) directly. Then the fluorescence was detected in GI tract, spleen and kidneys. Results showed that ZY-312 could be labeled by metabolic oligosaccharide engineering, and the optimal incubation time with AF647-DIBO was 5 h *in vitro*. Following oral gavage with AF647-DIBO labeled ZY-312 or AF647-DIBO alone, mice were subjected to *in vivo* imaging and the fluorescence intensity was similar in both groups 3 h, 6 h, and 12 h post the gavage. The fluorescence of AF647-DIBO group disappeared 24 h post gavage which was probably due to the excretion via GI tract. While the fluorescence of AF647-DIBO labeled ZY-312 retained in the cecum for as long as 48 h. Immunofluorescence assay further confirmed that labeled ZY-312 transiently colonized not only in cecum but also in stomach, ileum and colon of mice 48 h post-gavage and that no massive accumulation of ZY-312 was detected in other organs such as kidneys and spleen. In conclusion, ZY-312 could transiently colonize in GI tract, mainly in cecum, for at least 48 h, and it hardly disseminate to other organs, which shed new light on the future development of *B. fragilis* as a probiotic product.

## Introduction

The gut microbiota consists of around 2,000 different bacterial species (Boyce and Bertozzi, 2011), which are influenced by age, infection, and drugs, etc. Gut microbiota dysbiosis which is characterized by imbalance in the composition and function of these intestinal microbes is associated with a wide spectrum of diseases ranging from localized gastrointestinal disorders to neurologic, respiratory, metabolic, hepatic, and cardiovascular diseases (Lynch and Pedersen, 2016). Therefore, increased attention has been drawn on developing probiotics to help reestablish micro-ecological balance. *In-vivo* colonization is an important characteristic of probiotics, which means strains can survive *in vivo*, repair damaged intestinal mucosa and defend the invasion of pathogenic bacteria.(Deriu et al., 2013) Moreover, the ability of probiotics to colonize in intestine is considered to be a critical factor in immune regulation.(Lebeer et al., 2010) Therefore, many *in-vitro* models have been established to examine the adhesion and colonization ability of bacteria in different tissues and cell lines including partial intestinal tissue resected from human, mucus isolated from feces, and ileostomy effluent and cells such as HT-29 and Caco-2 (Ouwehand et al., 1995; Wang et al., 2018).

However, the models mentioned above did not mimic the mucus layer, which is the first barrier inoculated strains meet with. Mucin-secreting HT-29-MTX cells might overcome the shortcomings to some extent (Martín et al., 2015). Nevertheless, the composition of mucin produced by HT-29-MTX cells is different from that of mucin derived from the human intestinal mucosa. Moreover, current methods aimed to detect colonization of probiotics such as Gram staining, sequencing and fluorescent labeling still exists several limitations. First of all, similar strains cannot be distinguished by Gram staining. Secondly, sequencing is a time-consuming method. Conventional fluorescent labeling is useful but also limited, for fluorescent proteins require aerobic conditions, but most gut commensals are anaerobes (Craggs, 2009).

To overcome those limitations, we used metabolic oligosaccharide engineering (MOE) and bioorthogonal click chemistry (BCC) (Boyce and Bertozzi, 2011) to label and track live ZY-312. In this method, a small functional group is incorporated in biomolecules of the target strain by a cell’s endogenous biosynthetic function, and through BCC, this group reacts with a second chemical group, forming a stable covalent bond. (Sletten and Bertozzi, 2009) This method has been used to study glycoconjugates and polysaccharides in living systems, (Dehnert et al., 2011) including in prokaryotic organisms (Dumont et al., 2012; Kaewsapsak et al., 2013), *Staphylococcus aureus*, *Bacteroides ovatus*, etc.

In this study, we aimed to examine the colonization and dissemination of an emerging probiotic candidate ZY-312, a new strain of *Bacteroides. fragilis* through MOE and BCC method.

## Materials and Methods

### Mice

Male and female 6 to 8-week-old C57BL/6 mice were purchased from Beijing Vital River Laboratory. Mice were housed in SPF conditions with food and water *ad libitum*. The animal experimental procedures were performed in accordance with the Guide for the Care and Usage of Laboratory Animals published by the US National Institutes of Health (NIH Publication No.85–23, revised 1996), and approved by Nanfang Hospital Animal Ethics Committee (protocol # NFYY-2014-123). Mice were randomly assigned to three groups: AF647–DIBO labeled ZY-312 group, AF647-DIBO group, and negative control group. Mice in AF647–DIBO labeled ZY-312 group and AF647-DIBO group were inoculated with labeled strains or AF647–DIBO respectively, for various time periods, including 3 h, 6 h, 12 h, 24 h, 48 h, 60 h, 72 h, and 96 h. And negative control group didn’t receive any intervention.

### Bacterial Strains and Culture Media

*Bacteroides. fragilis* strain ZY-312 was provided from Zhiyi Biological Technology Co., Ltd. [Guangzhou, Guangdong province, (Deng et al., 2016; Wang et al., 2017)]. The strain was cultured under anaerobic conditions (80% N2, 10% H2, 10% CO2) in basal peptone-yeast broth with azide-modified tetraacetylated-N-azidoacetylgalactosamine (GalNAz) at a final concentration of 100 μM at 37°C in an anaerobic incubator (Bugbox, Ruskinn) during the whole study. Basal peptone-yeast broth contains (per liter) 5 g yeast extract, 20 g proteose peptone, 5 g NaCl, 5 mg hemin, 0.5 mg vitamin K1 and 5 g K_2_HPO_4_. Hemin, vitamin K1 and K_2_HPO_4_ were added through a filter after the basal medium had been autoclaved. Ten-fold serial dilutions of the cultures were plated on trypticase soya agar containing 5% fresh sheep blood to obtain single colony following microscopic examination (TSA; Oxoid, Basingstoke, United Kingdom).

### MOE Labeling of Bacteria

ZY-312 was cultured for 65 h at 37 °C under anaerobic conditions (80% N_2_, 10% H_2_, 10% CO_2_) in an anaerobic glove box in basal peptone-yeast broth (OXOID, United Kingdom) with GalNAz (Thermofisher, United States) at a final concentration of 100 μM. Bacterium were spun down and washed three times in 1 × phosphate-buffered saline (PBS) supplemented with 1% bovine serum albumin (BSA) when the OD_600_ value ranged from 0.7 to 1.4. The bacteria pellet was resuspended in 10 ml PBS supplemented with 1% BSA (Sigma, Germany) and 20 mM AF647-DIBO (100 μl per vial) and each vial of the bacterium suspension was kept separately. To determine the optimal incubation time, bacteria suspensions were incubated for 3 h, 5 h, 7 h, and overnight in dark on a shake flask. After incubation, bacteria were pelleted and washed five times with PBS supplemented with 3% BSA. Finally, the bacteria were resuspended either in PBS for *in vivo* tracing or in 90% glycerol for fluorescent microscope observation.

### *In vivo* Optical Imaging

Mice were divided into *B. fragilis* group and AF467-DIBO group and each group received gavage (300 μl) at different time point (*t* = -96 h, -72 h, -60 h, -48 h, -24 h, -12 h, -6 h, and -3 h) prior to the *in vivo* observation (three mice for each group and time point). Mice were caged separately according to the group and time point to avoid the intake of feces cross the group. For the *in vivo* observation, all mice were anesthetized with isoflurane followed by the *in vivo* imaging using Xtreme imaging system (Bruker) All mice were sacrificed after the *in vivo* imaging and the intestine, stomach, kidney and spleen were collected with adherent connective tissues resected. The distributions of the fluorescence-labeled ZY-312 in those organs were also determined through Xtreme imaging system. Image analysis was performed using Molecular Imaging Software version 7.2. A region of interest (ROI) was drawn around fluorescent signals of one representative mouse using the ellipse option in MI software. This ROI was then saved as template and applied to the other images.

### Frozen Sections

Tissue samples of different organs (three parallel samples for each organ) were collected in eppendorf tubes and washed with PBS to remove dirt and intestinal contents. Samples were dried on a clean filter and loaded on the pre-cooled embedding agent and covered with extra embedding agent. Cryosections were performed and by a freezing microtome and the slides were imaged on a microscope.

### Immunofluorescence

Slides were placed at room temperature for ten minutes and washed with PBS for three times (5 min per time) and dried with a filter paper. Then they were blocked with goat serum for 1 h at room temperature and incubated with actin antibody (diluted with 5%BSA and Triton at 1:250) in a humidified chamber overnight at 4°C. Subsequently, the slides were washed with PBS and incubated with FITC-conjugated secondary antibodies (Abcam) for 1 h at room temperature. Finally, the samples were covered with mounting medium containing DAPI and examined under laser confocal fluorescence microscopy.

### Imaging by Laser Confocal Fluorescence Microscopy

We respectively, chose red, blue, green filters of the confocal fluorescence microscopy to detect the fluorescence. FITC was detected through the green filter (excitation at 488 nm, emission at 525 nm), DAPI was through the blue filter (excitation at 340 nm, emission at 488 nm) and fluorescence of labeled ZY-312 was through red filter (excitation at 650 nm, emission at 665 nm).

### Statistical Analysis

Statistical analysis was done with Prism using unpaired Student’s *t*-test.

## Results

### *In vitro* Labeling of ZY-312 With AF647-DBIO

**FIGURE 1:**
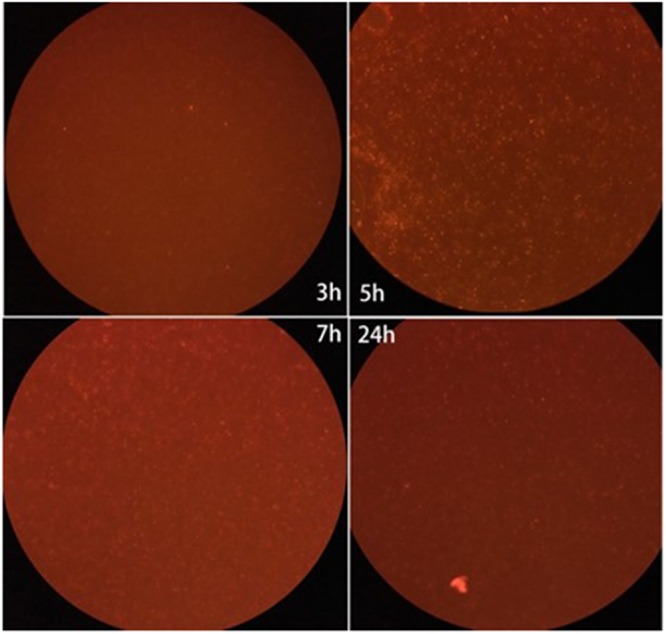
Fluorescence intensity of the labeled ZY-312 co-incubated with AF647-DIBO at various time points. ZY-312 was grown 65 h in basal peptone-yeast broth with GalNAz and the culture was co-incubated with AF647-DIBO for 3 h, 5 h, 7 h, and 24 h, respectively. When co-incubated for 5 h, Fluorescence intensity was the strongest among all time points.

To construct the MOE labeling of ZY-312, we incubated ZY-312 and AF647-DIBO with shaking and blocked from light for various time. Results showed that ZY-312 was amenable to AF647-DBIO labeling and the optimal incubation time could be 5 h (**Figure 1**).

### Visualization of Labeled ZY-312 *in vivo*

**FIGURE 2:**
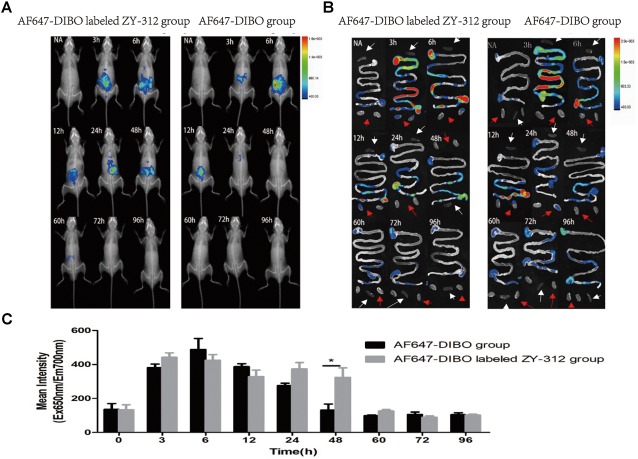
*In vivo* optical imaging of mice. **(A)** *In vivo* imaging of C57BL/6 mice in the AF647-DIBO group and the AF647-DIBO labeled ZY-312 group. **(B)** *In vivo* imaging of C57BL/6 mice’s GI tracts, spleens (white arrow) and kidneys (red arrow) in the AF647-DIBO group and the AF647-DIBO labeled ZY-312 group. **(C)** *In vivo* Mean Fluorescence Intensities of mice in AF647-DIBO group and AF647-DIBO labeled ZY-312 group. (^∗^*p* < 0.05) ROIs of similar areas were drawn around the fluorescent signals observed. Data are representative of at least three independent experiments.

We administered labeled ZY-312 to C57BL/6 mice by gavage and analyzed colonization of the bacteria in the GI tract. Generally, *in vivo* imaging showed that the fluorescence of both AF647-DIBO group and labeled ZY-312 group gradually declined over time. For AF647-DIBO administration group, the fluorescence could be detected at 3 h but faded away at 24 h and later, which might be attributed to the possibility that the dye should be metabolized or excreted completely after 24 h. To our surprise, labeled ZY-312 was detectable from 3 h to 48 h post-gavage, even longer than that of AF647-DIBO itself, indicating that ZY-312 was capable of colonizing in the GI tract for at least 48 h (**Figures 2A,C**).

To further evaluate the spatial distribution of labeled ZY-312, fluorescence of specific organs was imaged. As for the GI tract, fluorescence intensity of both groups was declined with time and fluorescence distribution in both groups was also similar in the first 12 h. At 3 h, the fluorescent signal was most prominent in the stomach and in middle and lower small intestine, and by 6 h it reached the cecum. Finally at 12 h, the fluorescence of both groups had gradually decreased and focused on the cecum and the colon, probably after which AF647-DIBO was excreted later owing to the absence of fluorescence throughout the GI tract at 24 h. However, there still existed fluorescent signal in the cecum of labeled ZY-312 treated mice at 24 h and even later at 48 h. Besides, no signal was detected in organs including the kidneys and spleens (**Figure 2B**).

Collectively, it was demonstrated that ZY-312 was likely to colonize in the GI tract, mainly in cecum, for at least 48 h.

### Imunofluorescence Assay of Labeled ZY-312 Colonizing in GI Tract

**FIGURE 3:**
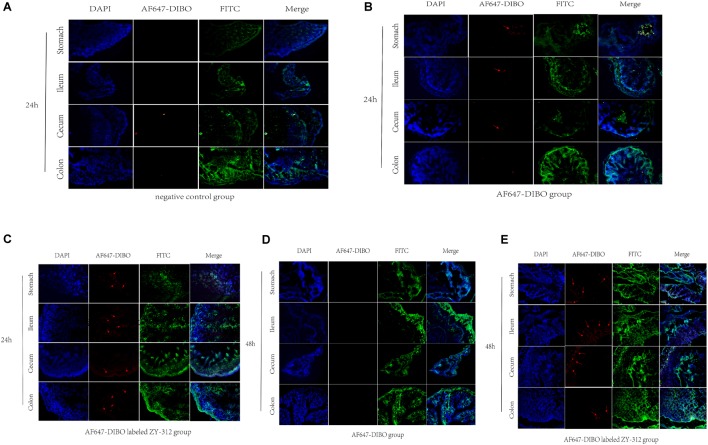
Imaging of mice using confocal microscopy **(A)** Immunofluorescence of C57BL/6 mice’s stomach, ileum. Cecum and colon in negative control group. **(B,C)** Immunofluorescence of C57BL/6 mice’s tissue in AF647-DIBO group **(B)** and AF647-DIBO labeled ZY-312 group **(C)** for 24 h after treatment. **(D,E)** Immunofluorescence in AF647-DIBO group **(D)** and AF647-DIBO labeled ZY-312 group **(E)** for 48 h after treatment. Data are representative of at least three independent experiments.

To further validate the colonization of ZY-312 in the GI tract, we observed the frozen sections of the excised stomach, ileum, cecum, and colon with immunofluorescence assay. Results showed that red fluorescence signal could be detected in the stomach, villi of ileum, cecum and colon only in AF647-DIBO labeled ZY-312 group but not in AF647-DIBO or negative control at 24 h and 48 h (**Figure 3**). Besides, no fluorescence signal was detected in the kidneys or spleen of mice gavaged with AF647-DIBO labeled ZY-312 or AF647-DIBO alone at 24 h and 48 h (data not shown).

## Discussion

Non-toxigenic *B. fragilis* is regarded as a probiotic candidate owing to its immunoregulatory property and other health-promoting benefits (Hsiao et al., 2013). Its protective effect is confirmed in mouse experimental colitis model (Mazmanian et al., 2005; Round and Mazmanian, 2010) and autoimmune encephalomyelitis model (Ochoarepáraz et al., 2010) by increasing the proportion of Foxp3^+^ regulatory T lymphocytes. It’s also capable to correct systemic immune defects in germ-free mice by stimulating maturation of splenic CD4^+^ T cells (Mazmanian et al., 2005). Recent study reveals that the tumors model of antibiotic-treated or germ-free mice do not respond to cytotoxic T-lymphocyte-associated protein 4 (CTLA-4) blockade treatment. However, the defect was overcomed by gavage with *B. fragilis*, immunization with *B. fragilis* polysaccharides, or adoptive transfer of *B. fragilis*–specific T cells, indicating that antitumor effect of CTLA-4 blockade is also associated with *B. fragilis* (Vétizou et al., 2015). ZY-312 is a new strain of non-enterotoxigenic *B. fragilis*, isolated from the feces of a healthy breast-fed infant. Previously we have demonstrated that ZY-312 possesses several probiotic properties, including tolerance to air, simulating gastric fluid (pH 3.0), intestinal fluid and bile (pH 6.8). It’s also capable of adhere to colon cells without cytotoxicity *in vitro*. (Deng et al., 2016) ZY-312 possess similar characteristics to standard *B. fragilis* strains ATCC 25285 in morphology, growth kinetics, metabolic, and genetic profile and has been proved to be non-toxigenic in both normal and immune-deficient mice (Wang et al., 2017). Furthermore, ZY-312 has been shown to be able to inhibit the growth and adhesion of *Vibrio parahaemolyticus* (*V. parahaemolyticus*) *in vitro* and protect both RAW 264.7 and LoVo cells from damage caused by *V. parahaemolyticus* (Li et al., 2017). Recently, we discovered the modulatory effect of ZY-312 on gut microbiota in rats, which contributes to the amelioration of antibiotic-associated diarrhea (Zhang et al., 2018). In general, ZY-312 has the potential to become the first probiotic representative of the dominant Bacteroidetes phylum.

The ability to colonize to gut is a crucial characteristic of probiotic. Recently, Geva-Zatorsky’s team revealed a new approach called MOE-BCC to label and track anaerobic commensals without affecting the viability, growth and carbohydrate metabolism of bacteria. Therefore, we adopted MOE-BCC to label and track ZY-312 in the host in real time. To track live ZY-312 *in vivo*, we incubated ZY-312 with DIBO derivatives, whose combination is copper-independent and is non-toxigenic to cells and bacteria. Our *in vivo* imaging study showed that labeled ZY-312 mainly distributed in stomach and ileum by 3 h, in cecum by 6 h and in cecum as well as colon by 12 h, which are roughly consistent with the distribution of *B. fragilis* reported in previous study (Gevazatorsky et al., 2015). Of note, while the AF647-DIBO alone was excreted via GI tract at 24 h, AF647-DIBO labeled ZY-312 transiently colonized in the cecum and colon for as long as 48 h. In addition, immunofluorescence assay convinced us that labeled ZY-312 transiently colonized not only in the cecum but also in other regions of GI tract including the stomach, ileum and colon in mice 48 h post-gavage, regardless of the possibility that the fluorescence might be weakened somewhat due to metabolism in gut. Taken together, we believe that ZY-312 is able to transiently colonize in GI tract, particularly in cecum, for at least 48 h. To test whether ZY-312 could colonize in the GI track for even longer, a more stable and sensitive labeling system is required.

Our experiments also suggest that oral administration of ZY-312 is relatively safe and feasible. As is shown, labeled ZY-312 was not detected in organs including the spleen and kidneys at 24 h and 48 h post gavage, suggesting that ZY-312 could be only limited in the GI tract, which minimizes the chances of bacterial translocation, bacteremia and other adverse effects. As probiotics do not enter the blood circulation, it is hard to perform regular pharmacokinetical study to determine their dosage and administration frequency. However, our results demonstrated that ZY-312 still massively concentrated in cecum and colon 24 h after administration, where gut microbiota are most abundant, and most of them were excreted after 48 h. Therefore, we speculate that a proper frequency for oral administration of live ZY-312 should be once to twice a day to create a stable and long-term colonization of ZY-312 in the cecum and colon, where it plays its role in regulating intestinal flora like other probiotics do, and could also prevent bacterial over-accumulation at the same time.

Nevertheless, our experiments still have some other limitations. Once given, the progenies of bacteria cannot be labeled by themselves due to the lack of azide-modified sugars in GI tract. Therefore, we can only investigate the migration and colonization of initial labeled ZY-312 but not the progeny strains. However, given that *B. fragilis* only makes up a very small part of the human intestinal commensal bacteria (Wang et al., 2017), it is unlikely that descendants of ZY-312 would be proliferate massively in the intestine.

In summary, our study shows that ZY-312 could transiently colonize in the GI tract, mainly in cecum, for at least 48 h, and that oral administration of ZY-312 is a relatively safe and feasible way for them to colonize in the large intestine and avoid its massive accumulation in GI tract and other organs which might do harm to health. Our work sheds new light on the development of *B. fragilis* as an ideal probiotic product for future clinical trial.

## Author Contributions

WX conducted the experiments with mice and wrote the manuscript. PS did the experiments with bacteria and contributed to revising the manuscript. LZ helped with performing the experiments and contributed to revising the manuscript. HF, YW, YYL, and YQL designed the experiments and contributed to revising the manuscript. FZ provided overall directions and contributed to revising the manuscript.

## Conflict of Interest Statement

The authors declare that the research was conducted in the absence of any commercial or financial relationships that could be construed as a potential conflict of interest. The reviewer YK and handling Editor declared their shared affiliation.
